# Editorial: Psychosocial Advances in Neuro-Oncology

**DOI:** 10.3389/fonc.2015.00243

**Published:** 2015-10-27

**Authors:** Tamara Ownsworth, Suzanne Kathleen Chambers, Haryana Mary Dhillon

**Affiliations:** ^1^Menzies Health Institute Queensland, School of Applied Psychology, Griffith University, Brisbane, QLD, Australia; ^2^Central Clinical School, Sydney Medical School, The University of Sydney, Sydney, NSW, Australia

**Keywords:** neuro-oncology, brain neoplasms, psychosocial distress, neuropsychology, supportive care

Neuro-oncology is a complex field encompassing scientific and clinical developments in the diagnosis and management of cancers directly affecting the central nervous system (CNS). These include brain tumors and metastases, and non-CNS cancers and treatments that produce neurocognitive impairment. To date, the dominant focus of neuro-oncology literature has been on the biological mechanisms and neurocognitive effects of brain tumor and cancer. However, neurocognitive impairments and psychological disorders arise from an interaction between physiological, medical, and psychosocial factors (1). Therefore, to guide holistic models of care, a biopsychosocial perspective is needed (2).

Psychosocial aspects of care focus on how people perceive and react to their diagnosis and symptoms and the ways in which they cope with their illness within their social context. Subjective reports of symptoms are often more closely related to quality of life than objective indices, such as neuropsychological test performance (2). High rates of depression and anxiety have been consistently reported in neuro-oncology samples, with distress found to persist or even increase over time (3). Due to the increased emphasis on outpatient care, family members assume the primary role in supporting individuals to cope with symptoms and the everyday impact of their illness. Cancer can place strain on relationships and compromise the physical and mental health of family members, in turn impacting their ability to provide sustained support to the person with cancer (4).

This Research Topic aims to enhance understanding of the neurocognitive and psychosocial consequences of neuro-oncological disorders. It also aims to showcase advances in supportive care and highlight future research priorities for this population.

The neurocognitive consequences of cancer were the focus of three articles. A meta-analysis by Ono and co-authors found overall evidence that adjuvant chemotherapy for breast cancer is associated with subtle cognitive impairment. To strengthen the evidence base, they recommended that future prospective longitudinal research examine cognitive impairment levels before and after chemotherapy, with comparisons made to pre-diagnosis functioning. Robinson and co-authors posed the question of whether screening tools, such as the Montreal Cognitive Assessment (MoCA), are sufficiently sensitive to the cognitive effects of brain tumor. Their findings suggested that a brief but tailored assessment may have greater sensitivity to detect mild or focal effects. Dwan and colleagues examined whether rates of cognitive impairment after brain tumor vary according to source of reference used (i.e., norms, controls, and premorbid functioning). Reassuringly, comparisons showed that rates of impairment were largely consistent across sources. They advocated for a multi-faceted neuropsychological test battery with a measure of estimated premorbid cognitive functioning to avoid over- or under-estimation of impairment.

Behavioral and social consequences of brain tumor were the topic of two articles led by Simpson and Ownsworth. Simpson and co-authors identified that rates of behavioral changes after brain tumor were variable based on both self-report (7–40%) and relative report (8–60%), and were higher for people with seizures and poorer functional status. Routine assessment and multi-level management of behavioral concerns was recommended. Qualitative research by Ownsworth and co-authors investigated family caregivers’ experiences of support and relationship changes. Due to the many issues found to impact on caregiver perceptions, it was recommended that professionals explore caregivers’ expectations and preferences for support throughout the illness.

Psychological well-being after brain tumor was the key theme of two articles. Trad and colleagues used the Distress Thermometer to screen for distress in different groups of patients and caregivers at initial diagnosis and tumor recurrence. The high rate of distress in the patient and caregiver groups at both time points underscores the key role of neuro-oncology care coordinators in providing access to psychosocial support throughout the care continuum. In their perspective article, Ownsworth and Nash emphasized the importance of assessing existential well-being or people’s sense of meaning, purpose, and value in life, in addition to mood and distress levels. Different avenues of existential support were discussed for facilitating the meaning making process across the illness trajectory.

Supportive care interventions for the neuro-oncology population were a focus of five articles, including two intervention studies. Jones and co-authors piloted a telephone-based psychological support intervention for people with brain tumor. The results of their single-case research provided preliminary support for the feasibility and utility of tele-based therapy for enhancing mental health and quality of life. A larger controlled trial is needed to examine factors influencing the efficacy of tele-based therapy. King and Green evaluated the efficacy of group cognitive rehabilitation for cancer survivors in a randomized controlled trial. Their findings generally supported the efficacy of their group intervention, with gains most apparent for perceived cognitive impairment.

In a systematic review of interventions to improve information provision for brain tumor patients, Langbecker and Janda found that most studies reported high rates of satisfaction with information provision. However, few examined improvements in knowledge and the methodological quality was generally low. A scoping review of psychotherapy interventions by Kangas similarly highlighted the paucity of evidence-based interventions for managing anxiety and depressive symptoms for this population. Cormie and colleagues considered the potential for exercise interventions to counteract the broader consequences of cancer, including fatigue, cognitive impairment, depression, and anxiety. Their perspective article discusses the benefits of targeted exercise programs for patients with CNS cancers and the need for research that examines both safety and efficacy of interventions.

In the final article of this Research Topic, Chambers and colleagues present an overview of the challenges and strategies for integrating quality standards of psychosocial care into neuro-oncology. They call for the development of a comprehensive model of survivorship care for people affected by brain tumor and their families.

Overall, the development and evaluation of psychological and supportive care interventions for people with neuro-oncological illness is an area of emerging research and of high interest to health professionals working in the field. International quality standards stipulate the need for cancer care facilities to provide assessments of patient distress and appropriate interventions (5). This practical and evidence-based text provides a unique and timely resource on the psychosocial care needs of people with neuro-oncological conditions and emerging intervention approaches.

## Author Contributions

All editors contributed to the Editorial, including preparing and editing the draft and approved the final version.

## Conflict of Interest Statement

The authors declare that the research was conducted in the absence of any commercial or financial relationships that could be construed as a potential conflict of interest.
